# Estimating the impact of universal antiretroviral therapy for HIV serodiscordant couples through home HIV testing: insights from mathematical models

**DOI:** 10.7448/IAS.19.1.20864

**Published:** 2016-05-11

**Authors:** Sarah T Roberts, Aditya S Khanna, Ruanne V Barnabas, Steven M Goodreau, Jared M Baeten, Connie Celum, Susan Cassels

**Affiliations:** 1 Department of Epidemiology, University of Washington, Seattle, WA, USA; 2 Department of Global Health, University of Washington, Seattle, WA, USA; 3 Department of Medicine, University of Chicago, Chicago, IL, USA; 4 Department of Medicine, University of Washington, Seattle, WA, USA; 5 Fred Hutchinson Cancer Research Center, Seattle, WA, USA; 6 Department of Anthropology, University of Washington, Seattle, WA, USA; 7 Department of Geography, University of California Santa Barbara, Santa Barbara, CA, USA

**Keywords:** HIV serodiscordant couples, antiretroviral therapy, HIV incidence, mathematical modelling, agent-based stochastic network modelling, exponential random graph models (ERGMs)

## Abstract

**Introduction:**

Antiretroviral therapy (ART) prevents HIV transmission within HIV serodiscordant couples (SDCs), but slow implementation and low uptake has limited its impact on population-level HIV incidence. Home HIV testing and counselling (HTC) campaigns could increase ART uptake among SDCs by incorporating couples’ testing and ART referral. We estimated the reduction in adult HIV incidence achieved by incorporating universal ART for SDCs into home HTC campaigns in KwaZulu-Natal (KZN), South Africa, and southwestern (SW) Uganda.

**Methods:**

We constructed dynamic, stochastic, agent-based network models for each region. We compared adult HIV incidence after 10 years under three scenarios: (1) “Current Practice,” (2) “Home HTC” with linkage to ART for eligible persons (CD4 <350) and (3) “ART for SDCs” regardless of CD4, delivered alongside home HTC.

**Results:**

ART for SDCs reduced HIV incidence by 38% versus Home HTC: from 1.12 (95% CI: 0.98–1.26) to 0.68 (0.54–0.82) cases per 100 person-years (py) in KZN, and from 0.56 (0.50–0.62) to 0.35 (0.30–0.39) cases per 100 py in SW Uganda. A quarter of incident HIV infections were averted over 10 years, and the proportion of virally suppressed HIV-positive persons increased approximately 15%.

**Conclusions:**

Using home HTC to identify SDCs and deliver universal ART could avert substantially more new HIV infections than home HTC alone, with a smaller number needed to treat to prevent new HIV infections. Scale-up of home HTC will not diminish the effectiveness of targeting SDCs for treatment. Increasing rates of couples’ testing, disclosure, and linkage to care is an efficient way to increase the impact of home HTC interventions on HIV incidence.

## Introduction

A key challenge for HIV prevention is delivering effective and efficient interventions to populations at highest risk of HIV acquisition, such as HIV-negative persons in stable, ongoing relationships with HIV-positive sexual partners, i.e. serodiscordant couples (SDCs). Although there is debate about the proportion of new infections that occur within SDCs [1–4], even the most conservative estimates suggest that an average of 29% of new infections across sub-Saharan African countries occur within this group [4]. To address this risk, the World Health Organization (WHO) recommends universal antiretroviral therapy (ART) initiation for all HIV-positive members of SDCs, regardless of CD4 count, to reduce the risk of transmission to their partners [5]. However, many countries have not incorporated these recommendations into national ART guidelines [6], and in countries that have, the uptake of ART among SDCs has been low [7,8].

Universal ART for SDCs could have considerable impact on population-level HIV incidence if it is delivered at high coverage levels [9]. Although the WHO now recommends universal ART initiation for all HIV-positive persons, achieving this target will be difficult for many countries due to resource constraints and implementation challenges. Countries will need to make strategic choices around how best to use ART to maximize prevention and treatment goals [10]. Because they are at higher risk of transmitting HIV, targeting SDCs for ART may have a larger impact on HIV incidence than scale-up of ART in the general population, and may be an efficient way to maximize prevention efforts.

In order for universal ART for SDCs to be an effective strategy, however, SDCs must be identified through couples’ HIV testing and counselling (HTC) interventions. Uptake of couples’ HTC has been low in Africa (10–20%) [11–14], despite recommendations to incorporate it into existing HTC programmes [15]. Implementation science research has identified strategies to markedly increase testing and linkage to care in the general population, including home HTC campaigns [16,17]. In these campaigns, although testing was limited to couples residing together in households, testing uptake is as high as 80% [16], and among couples tested together, disclosure rates are more than 95% [11]. Home HTC campaigns with strategies to reach partners living apart could also increase the rates of couples’ HTC, which could improve the identification of SDCs and thus increase ART initiation and viral suppression [18]. Alternatively, because these campaigns have achieved high rates of testing and linkage to care in the general population, little may be gained by targeting SDCs. For policy makers, it is important to estimate the magnitude of the benefit of universal ART for SDCs when it is offered in addition to programmes to increase ART uptake for HIV-positive individuals regardless of their partner's status.

We conducted a mathematical modelling study to estimate the impact of universal ART for SDCs in addition to ART scale-up through home HTC in two regions: KwaZulu-Natal (KZN), South Africa, and southwestern (SW) Uganda. Both regions have a higher HIV prevalence than the rest of the country, but they differ substantially with respect to absolute HIV prevalence (28% and 10%, respectively), sexual behaviour characteristics, and the proportion of stable couples who are HIV serodiscordant [19–24]. The primary outcome was the reduction in HIV incidence over a 10-year period. Secondary outcomes included the cumulative proportion of HIV infections averted and the proportion of HIV-positive persons who were virally suppressed at the end of the intervention period.

## Methods

To simulate HIV transmission in KZN, South Africa, and SW Uganda, we created dynamic, stochastic agent-based network models, parameterized using demographic, biological, behavioural, and treatment data. The models were derived from the exponential-family random graph modelling (ERGM) framework, and programmed using the statnet suite of packages in the R programming language [25]. These tools have been used to model HIV transmission and interventions in a number of settings [26–29].

Our analyses build on previous work that estimated the impact of the WHO guidelines on prevention of mother to child transmission (PMTCT) on adult HIV incidence (“PMTCT model”) [22]. Additional model features were added to explicitly include modelling of ART for SDCs. Parameters and computer code to reproduce our results are publicly available in a GitHub repository [30].

### Data

Our primary data source was a prospective study of home HTC to improve testing and linkage to care conducted from 2011 to 2012 [21]. Home HTC was offered to consenting adults (18 years or older) in defined geographic regions in the Vulindlela district of KZN and Mbarara district of SW Uganda. A total of 1,272 individuals in South Africa and 2,121 individuals in Uganda were tested. The study also included a community survey (*n*=268 in KZN, *n*=232 in SW Uganda). The study provided model parameters for sexual network characteristics, including the momentary (cross-sectional) distribution of the number of partnerships, the start and end dates of the three most recent partnerships, the ages of sexual partners, and ART uptake and viral suppression at study baseline. The sexual network data from the empirical study represented longer term primary and casual heterosexual partnerships. Additional model parameters were drawn from the published literature, and are described in detail in the Web Appendix (Supplementary File 1).

### Baseline model

As described previously, baseline HIV epidemics were simulated to capture existing epidemic features among adults (aged 18 to 55 years) in KZN and SW Uganda, including ART coverage levels [22]. Our models were calibrated using published HIV incidence [31,32] and prevalence [20,33] estimates.

Each setting was populated with 5,000 individuals at the start of the dynamic simulations, with age and sex distributions derived from published estimates [34]. Each simulated time step equalled 14 days. The following process were explicitly modelled: (1) mortality (all-cause mortality, plus CD4-based for HIV-positive individuals), (2) entry into population at age 18 (set to ensure growth rates consistent with census data for both regions), (3) pregnancy (based on age-specific fertility rates), (4) the formation and dissolution of partnerships (set to maintain mean number of partnerships and cross-sectional distribution of partnership counts consistent with empirical data), (5) temporal evolution of CD4 count and viral load (both modelled as functions of time since infection, and adjusted for ART status, which was derived from clinical data), (6) update of ART status and (7) the transmission of HIV (from HIV-positive to HIV-negative individuals, with probabilities derived from viral load and stage of infection of infected individuals). These processes are described in greater detail in the Web Appendix (Supplementary File 1).

In the baseline model, ART uptake was set equal to 53% in KZN and 48% in SW Uganda [21], with a mean CD4 count at ART initiation of 100 cells/µl in KZN [35] and 131 cells/µl in SW Uganda [36]. Adherence was estimated by the proportion of individuals on ART who were virally suppressed: 85% in KZN and 88% in SW Uganda [21]. Coverage was calculated as the product of uptake and adherence: 45% in KZN and 43% in SW Uganda. These coverage levels determined the proportion of HIV-positive individuals who accessed ART for treatment in the baseline model. In addition, pregnant women accessed HIV testing through antenatal care at 22 weeks gestation, with a 57% [23] uptake of ART for PMTCT among HIV-positive pregnant women in Uganda and 89% [24] in South Africa. Based on national guidelines at the time of the model design, we assumed that, in both countries, pregnant women with CD4 counts equal to or greater than 350 cells/ml received lifelong ART, and pregnant women with CD4 counts greater than 350 cells/ml received zidovudine (AZT) from the first antenatal visit until delivery, followed single-dose nevirapine during labor and AZT plus lamivudine during labor and for one-week postpartum (WHO Option A). As with ART for treatment, we calculated PMTCT coverage as the product of uptake and adherence. We assumed 75% adherence to PMTCT in this population [25].

### Model scenarios

We compared three scenarios, each simulated over a 10-year period from the end of the baseline simulation (Table 1). In the “Current Practice” scenario, the baseline model was extended for 10 years to estimate the trajectory of the epidemics in the absence of new interventions. We did not model ART initiation at higher CD4 counts in this scenario, despite recent changes in ART eligibility criteria, because a recent meta-analysis suggests that previous changes in ART eligibility guidelines have not resulted in increases in CD4 count at ART initiation [37].

**Table 1:** Key parameters for each model scenario

Scenario	Parameters	KZN, South Africa	SW Uganda
***Current Practice scenario***	ART uptake	53%	48%
Describes routine ART delivery in	CD4 count for ART eligibility	<350 cells/µl	<350 cells/µl
the general population	CD4 count at ART initiation	100 cells/µl	131 cells/µl
	Adherence	85%	88%
	ART coverage (uptake * adherence)	45%	43%
***Home HTC scenario***	Campaign frequency	Every three years	Every three years
Current Practice scenario plus home	Uptake of HIV testing	80%	80%
HTC campaigns	ART uptake among eligible HIV-positive persons	58.4%	58.4%
	Reduction in unprotected sex among known stable SDCs	63%	63%
***ART for SDCs scenario***	CD4 count for ART eligibility in SDCs	Universal (no threshold)	Universal (no threshold)
Home HTC scenario plus delivery of universal ART for stable SDCs during home HTC campaigns	ART uptake among known stable SDCs	90%	90%

In the Current Practice scenario, the ART coverage conditions simulated during the baseline model are continued with no additions or modifications. These same conditions persist in the Home HTC and ART for SDCs scenarios, but additional interventions are modelled in addition to routine ART delivery.

In the “Home HTC” scenario, we simulated a home HTC campaign every three years (at the start of years 1, 4, 7 and 10), based on previous modelling results [38]. Based on two recent systematic reviews of interventions for HIV testing and linkage to care in sub-Saharan Africa [16,17], we estimated 80% uptake of HIV testing through home HTC programmes. Among those who received testing and were eligible for ART (based on eligibility criteria of CD4 less than 350 cells/µl), 58.4% initiated ART [16], and initiation occurred immediately after testing.

SDCs were identified from the perspective of HIV-positive persons: if their longest-running partnership was with an HIV-negative person, they were considered to be part of an ongoing, stable SDC. A couple was considered a “known” SDC if both members of the SDC were tested during the home HTC campaign. Disclosure between partners occurred 100% of the time if both partners tested, consistent with nearly universal disclosure by month 12 in a recent study of home HTC [11]. In the Home HTC scenario, known SDCs were not offered universal access to ART regardless of CD4 count, based on national policy in South Africa and Uganda at the time of the modelling [39]. However, we modelled a 63% reduction in the frequency of unprotected sex in known stable SDCs based on self-reported data on the effect of couples testing and disclosure [40].

The “ART for SDCs” scenario replicated the Home HTC scenario, with the addition that an HIV-positive person was eligible for ART initiation, regardless of CD4 count, if she or he tested during home HTC and was identified as a member of a known SDC. ART uptake was modelled at 90% among HIV-positive members of known SDCs [41]. In a sensitivity analysis, we evaluated the impact of our assumption of a 63% reduction in unprotected sex after couples testing, by repeating the Home HTC scenario with no change in unprotected sex among known SDCs.

### Outcomes

We simulated each scenario ten times to account for statistical variation, consistent with other network modelling studies [26,27]. The primary outcome was the adult HIV incidence rate 10 years after the start of implementation, averaged over ten model simulations. We computed 95% confidence intervals using a theoretical *t*-distribution, defined as $\bar{X}\pm t_{df=(n-1),0.975\frac{\sigma }{\sqrt{n}}}^{*}$, where *X* is the random variable of interest, *n* is the number of simulation runs, and σ is the standard deviation. These confidence intervals only capture variation across simulations, not variation due to changing parameter values.

Secondary outcomes were the cumulative proportion of HIV infections averted over the 10 years of intervention, and the proportion of all HIV-positive individuals (including treated and untreated individuals in the denominator) who were virally suppressed (viral load ≤100 copies/mL) at the end of the 10th year.

## Results

At the end of the baseline simulation, HIV prevalence was approximately 25% in KZN and 10% in SW Uganda. HIV incidence was 2.31 per 100 person-years (py) in KZN and 0.83 per 100 py in SW Uganda. These parameters were consistent with empirical data (Table 2). The modelled populations also showed high agreement with other empirical parameters, in particular the proportion of HIV-positive persons who are in SDCs, the proportion of all stable partnerships that are serodiscordant, and the proportion of all HIV-positive persons who are virally suppressed (Table 2).

**Table 2:** Comparison of model characteristics and empirical data

	KZN, South Africa		SW Uganda	
		
	Model estimates	Empirical data	Model estimates	Empirical data
HIV prevalence	25%	28%^a^ [19]	10%	10%^a^ [20]
HIV incidence (per 100 person-years)	2.31	2.20^a^ [32]	0.83	0.80^b^ [42]
Proportion of couples who are serodiscordant	17%	21%^a^ [23]	6%	6%^b^ [24]
Proportion of HIV-positive persons in stable SDCs	32%	31%^a^ [23]	29%	26%^b^^c^ [24]
Proportion of HIV-positive persons who are virally suppressed	29%	25%^b^ [43]	28%	22%^a^^d^ [24]

a Regional-level data

b National-level data

c Calculated using the methods presented in Chemaitelly *et al*. 2012. [44]

d Proportion of HIV-positive persons who are on ART. Best available country-specific estimate of viral suppression.

**Figure 1:**
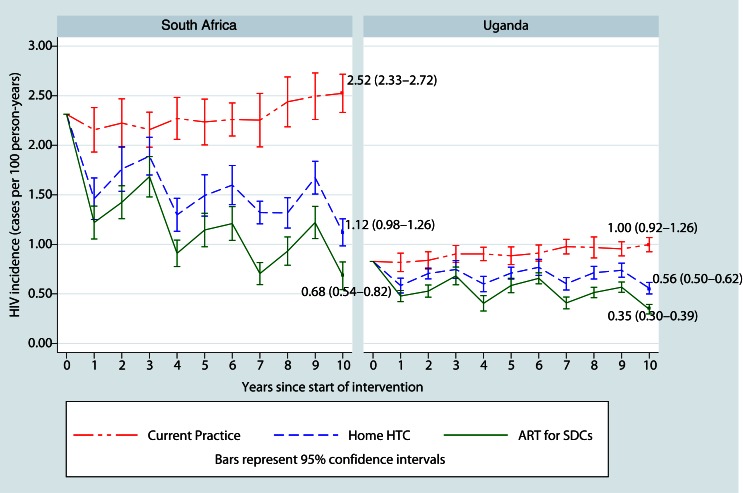
Mean HIV incidence (cases per 100 person-years) over 10 years of implementation in each scenario.

Figure 1 summarizes HIV incidence in each scenario. In the 10th year, mean HIV incidence in KZN was 2.52 per 100 py (95% confidence interval [CI]: 2.33–2.72) in the Current Practice scenario and 1.12 cases per 100 py (95% CI: 0.98–1.26) in the Home HTC scenario, a reduction of 1.4 cases per 100 py (56%). In SW Uganda, mean HIV incidence was 0.44 per 100 py (44%) lower in the Home HTC scenario (mean HIV incidence 0.56, 95% CI: 0.50–0.62) than in the Current Practice scenario (mean HIV incidence 1.00, 95% CI: 0.92–1.07). The effects of home HTC varied over time in both settings: the largest reduction in incidence was achieved by the first HTC campaign in year 1, after which there was a cyclical rise and fall with subsequent rounds of intervention at the start of years 4, 7 and 10 (Figure 1). In our sensitivity analysis, there was no change in mean HIV incidence when we eliminated the 63% reduction in unprotected sex among stable SDCs in the Home HTC scenario.

In both settings, the ART for SDCs scenario resulted in lower incidence than the Home HTC scenario (Figure 1). In KZN, the addition of universal ART for SDCs resulted in a mean HIV incidence in the 10th year of 0.68 per 100 py (95% CI: 0.54–0.82), 0.44 per 100 py (39%) lower than in the Home HTC scenario. In SW Uganda, HIV incidence was 0.21 per 100 py (38%) lower, with mean incidence of 0.35 per 100 py (95% CI: 0.30–0.39).

**Figure 2:**
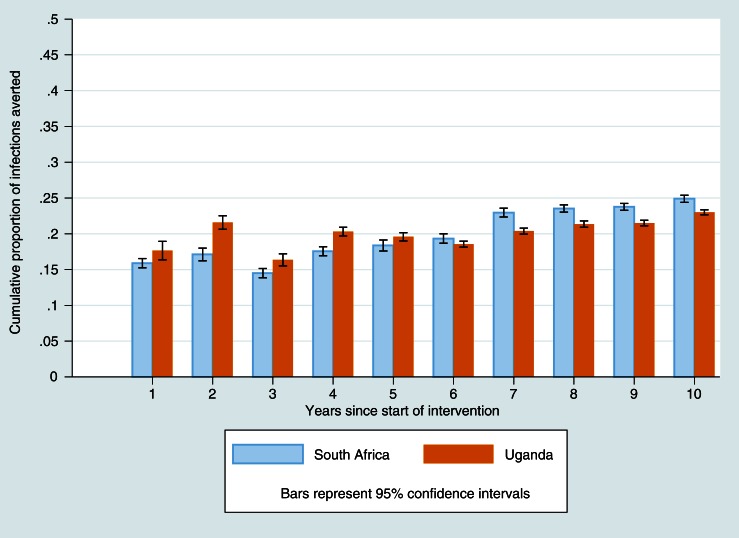
Cumulative proportion of HIV infections averted over time with ART for SDCs compared to Home HTC.

Because incidence varied cyclically in both the Home HTC and ART for SDCs scenarios, we examined an additional measure of effect, the cumulative proportion of infections averted over time in the former scenario compared to the latter. Results are shown in Figure 2. Adding universal ART for SDCs to home HTC averted 16% of new HIV infections after the first year in KZN, and 18% in SW Uganda. By the end of year 10, the proportion of infections averted increased to 25% in KZN and 23% in SW Uganda.

**Figure 3:**
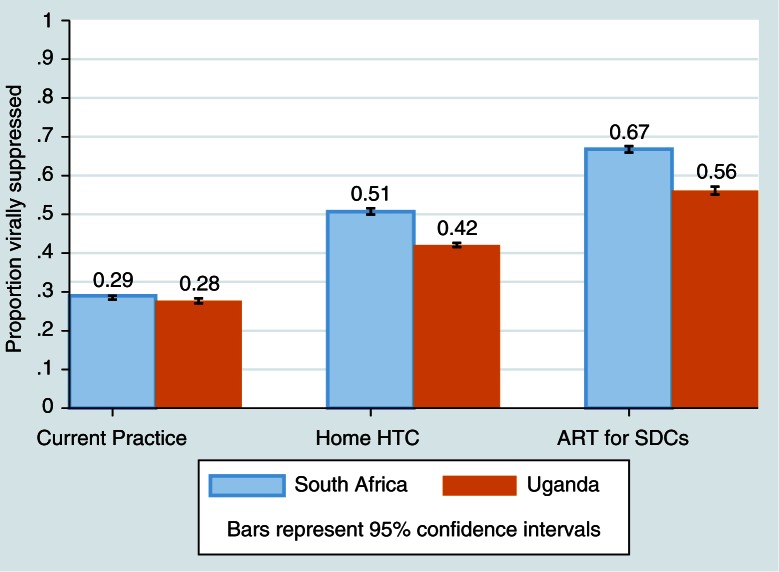
Proportion of HIV-positive persons virally suppressed at the end of 10 years of each model scenario.

In KZN, the proportion of HIV-positive persons who were virally suppressed increased from 29% in the Current Practice scenario to 51% with Home HTC, and to 67% in the ART for SDCs scenario. In SW Uganda, 28% were virally suppressed in the Current Practice scenario, 42% in the Home HTC scenario, and 56% in the ART for SDCs scenario (Figure 3).

## Discussion

Our findings suggest that a home HTC and linkage to care intervention can substantially lower HIV incidence in KZN and SW Uganda, consistent with previous modelling studies in KZN [45,46]. Furthermore, we found that a combination prevention intervention that provided universal ART for stable SDCs in the context of home HTC was even more impactful, reducing HIV incidence by about 38% over home HTC alone. The effect of the intervention was greatest in the years of HTC campaigns, after which incidence slowly increased until the next campaign; due to this cyclical nature, the proportion of infections averted by the universal ART for SDCs intervention was less than the reduction in incidence at year 10. Yet both outcome measures suggest substantial reductions in HIV incidence, complementing the home HTC intervention. Combining universal ART for SDCs with home HTC brought both populations closer to the UNAIDS target of 73% viral suppression among HIV-positive persons by the year 2020 [47].

Over a 10-year period, universal ART for SDCs increased the proportion of virally suppressed HIV-positive persons by only 15%, but averted 24% of new infections, relative to the home HTC intervention. This enhanced effectiveness can be attributed to the fact that the intervention was targeted to persons known to be at risk of transmitting HIV to an uninfected partner. In contrast, a recent observational study found that a 1% increase in ART coverage in the general population corresponded to a 1.4% decrease in the risk of HIV infection [32]. Taken together, these findings imply that targeting ART to a high-risk population creates a larger impact on HIV incidence than a scale-up in the general population. In addition, our work suggests that home HTC can be an effective way to identify stable SDCs, in order to attain high coverage of this intervention.

Only one previous model has assessed the impact of ART for stable SDCs on population-level HIV incidence. El-Sadr and colleagues [9] found that, at 80% coverage, this intervention would result in substantial reductions in HIV incidence, ranging from 16–40% in a country with high HIV prevalence (Lesotho) or 17–37% in a country with moderate HIV prevalence (Malawi). However, the reductions in incidence estimated by El-Sadr *et al*. are relative to a scenario with no ART scale-up in the general population, and their model does not account for the effects of ART for stable SDCs on reductions in HIV transmissions to concurrent sexual partners, or subsequent “downstream” effects on new partnerships. By accounting for these additional benefits of ART for stable SDCs, we estimate a greater impact of this intervention, and show that it still substantially reduces HIV incidence even when paired with strategies to scale up ART coverage in the general population.

One strength of this work is that we used individual- and partner-level data from a study of a highly successful home HTC intervention that resulted in high rates of HIV testing, linkage to care and viral suppression [21]. Our agent-based network model is novel because it incorporates the effects of partnership dynamics in the estimation of population-level intervention effectiveness. Previous studies support the notion that both concurrent partnerships and relationship duration impact HIV transmission [48–50]. The interaction between the intervention and these partnership dynamics may explain why we saw similar reductions in HIV incidence in the two settings, despite differences in epidemic characteristics and sexual behaviour patterns. For example, in SW Uganda, rates of concurrency and partnership dissolution are lower than in KZN; ART initiation is highly protective for the HIV-negative partner within a partnership, but there may be fewer infections averted within concurrent or subsequent partnerships. On the other hand, KZN has higher concurrency rates and shorter partnership duration. Thus, HIV-negative partners may have a higher risk of acquisition from concurrent partnerships, but there is also a greater potential for ART initiation in the HIV-positive partner to prevent HIV transmission in sexual relationships formed after ART initiation. This highlights the importance of using transmission models that explicitly consider sexual network structures.

Our model also allowed for a unique approach to identifying stable SDCs to determine eligibility for universal ART for SDCs. This intervention is designed to target SDCs in stable, long-term relationships, because people in short-term or casual relationships would be unlikely to utilize couples-based HIV testing or treatment [4,51,52]. Many previous mathematical models have defined “stable” SDCs as cohabiting couples, and used data from Demographic and Health Surveys or other published sources to parameterize the proportion of the population who are stable SDCs [1–3,50,53]. We used the self-reported sexual behaviour patterns and network-based modelling of HIV transmission to simulate SDCs within the model population, instead of explicitly including a parameter to define the proportion of SDCs. We considered an SDC to be stable if it was the only partnership or the longest-running partnership, from the perspective of the HIV-positive partner. Our model matched very closely with empirical estimates on the proportion of HIV-positive persons who are in stable SDCs, indicating that this was a reasonable approach.

Several limitations of this study should be noted. The model did not include short-term or “one-off” partnerships due to the limitations in the data. We may therefore have overestimated the proportion of HIV transmission occurring within stable SDCs and the impact of the ART for SDC intervention. However, our estimate of the proportion of HIV-positive persons in stable SDCs is consistent with empirical data, and data from our parent study and other published literature indicates that only a small proportion of people are involved in multiple short-term partnerships [21,24,54]. We did not model increased rates of relationship dissolution in SDCs, although studies indicate that known SDCs have higher rates of separation than couples with concordant status or unknown HIV status [55–58]. Because separation decreases the risk of HIV transmission within that couple, primarily when the HIV-positive partner is not on ART, our assessment of the impact of universal ART for SDCs on reducing HIV incidence may be overestimated [50]. Data on couples’ testing dynamics were not available, so we assumed testing behaviour of partners in stable relationships was independent. This implies that the probability of both partners testing together was 64%. If the true proportion was higher, the home HTC intervention could identify a larger number of SDCs and have a greater impact on HIV incidence. We did not model the concurrent scale-up of ART for treatment or PMTCT over the 10-year intervention period, including the adoption of current WHO recommendations for lifelong ART for pregnant women (WHO Option B+) in both countries. Although such scale-up of alternate strategies for treatment as prevention would have an effect on absolute HIV incidence, it is not clear how much coverage can realistically improve without targeted strategies such as home HTC campaigns [37]. In a previous modelling study, we found that adoption of Option B+ at current PMTCT coverage levels could result in a relative 15% decrease in HIV incidence in KZN and SW Uganda [22]. Future work should evaluate the joint impact of universal ART for both pregnant women and SDCs in these settings. Finally, we did not account for the costs of ART scale-up or home HTC for SDCs. However, a recent study showed that home HTC is a cost-effective strategy for HIV prevention [45], and the addition of universal ART for SDCs is likely to be even more cost-effective.

## Conclusions

Universal ART for SDCs has the potential to substantially reduce HIV incidence in the context of ART scale-up in the larger community. The critical implementation challenge is to ensure that both members of a couple are tested for HIV during HTC campaigns and that they receive post-test counselling and access to ART. Implementation science studies evaluating strategies to increase uptake of ART among SDCs will provide insight on the best way to fill this gap. It is important for policymakers to know that the effects of targeting SDCs for ART are retained when ART is also scaled-up in the general population. Thus, providing universal ART to SDCs is actually additive to home HTC and likely to be higher-yield, with a lower number needed to treat than HTC alone.

## Supplementary Material

Estimating the impact of universal antiretroviral therapy for HIV serodiscordant couples through home HIV testing: insights from mathematical models
